# Combined targeting of EGFR/HER promotes anti-tumor efficacy in subsets of KRAS mutant lung cancer resistant to single EGFR blockade

**DOI:** 10.18632/oncotarget.3853

**Published:** 2015-05-15

**Authors:** Ijeoma Adaku Umelo, Olivier De Wever, Peter Kronenberger, Jan Van Deun, Alfiah Noor, Kshitiz Singh, Erik Teugels, Gang Chen, Marc Bracke, Jacques De Grève

**Affiliations:** ^1^ Laboratory of Molecular Oncology and Department of Medical Oncology, Oncologisch Centrum, Universitair Ziekenhuis Brussel, Brussels, Belgium; ^2^ Laboratory of Experimental Cancer Research and Department of Radiotherapy, Universitair Ziekenhuis Gent, Brussels, Belgium; ^3^ Laboratory of Biotechnology, Department of Healthcare, Erasmushogeschool Brussel, Brussels, Belgium; ^4^ Laboratory of Gene Therapy & Regenerative Medicine, Vrije Universiteit Brussel, Brussels, Belgium

**Keywords:** lung cancer, KRAS, EGFR, HER, targeted therapy

## Abstract

KRAS is a frequently mutated oncogene in lung cancer and among the most refractory to EGFR targeted therapy. Recently, preclinical evidence in pancreatic cancer has demonstrated that mutant KRAS can be regulated by EGFR. However, the distinct correlation between the EGFR/HER family members and mutant KRAS has not been investigated. Here, we show that non-small cell lung cancer cell lines harboring differing isoforms of mutant KRAS, can be broadly divided into EGFR/HER dependent and EGFR/HER independent groups. Combined therapeutic targeting of EGFR, HER2 and HER3 in isoforms regulated by extracellular growth signals promotes *in vitro* and *in vivo* efficacy. We also provide evidence that depletion of EGFR via RNA interference specifically abolishes the EGFR/KRAS interaction in the dependent subset. Taken together, these findings suggest that upstream inhibition of the EGFR/HER receptors may be effective in treating a subset of KRAS mutant lung cancers.

## INTRODUCTION

The identification and characterization of genetic abnormalities attributed to the development of lung cancer has enabled advancement in understanding the biology and pathophysiology of the disease. The most common genetic alterations observed in non-small cell lung cancer (NSCLC) occur in the epidermal growth factor receptor (EGFR) gene, a member of the HER family (Human Epidermal Growth Factor Receptor) of transmembrane receptor tyrosine kinases (RTKs), and in KRAS - a member of the Ras family of small GTPases, which also includes HRAS and NRAS [1]. Oncogenic KRAS missense mutations are found in approximately 10–30% of lung carcinomas where they typically cluster around codon 12, codon 13 or more rarely codon 61 [2–3]. These substitutions have been described to deregulate RAS signaling by decreasing GTP-ase activity, and hence constitutively activating down-stream signaling molecules independent of ligand-mediated EGFR activation [4]. Current knowledge suggests that this downstream activation renders upstream EGFR inhibition irrelevant in the context of possible therapeutic intervention. Moreover these cancers are consistently wild-type EGFR which on itself precludes significant therapeutic efficacy from single EGFR inhibition.

There is currently no established therapy available for KRAS mutant cancers [5]. Various efforts to specifically target KRAS with farnesyl transferase inhibitors (FTIs), which block RAS membrane attachment and RAS signaling, have failed to show a significant enzyme inhibitory activity needed for clinical activity, which may explain the limited therapeutic effect of FTIs in phase II lung cancer trials [6]. Other strategies to specifically target the activity of oncogenic RAS have also provided no significant therapeutic benefit, although this still remains an active area of research [7–8]. Currently, downstream targeting of the RAS pathway (MEK inhibition) is under clinical investigation [9–11].

While multiple reports have described the constitutively active oncogenic KRAS to be independent of EGFR [12–13], recent evidence in pancreatic cancer has indicated that signaling by mutant KRAS may be dependent on upstream activation proposed of EGFR [14–15]. Moreover, Young et al, recently proposed that oncogenic KRAS may also be regulated by upstream activation of several receptor tyrosine kinases (RTKs) [16]. Besides EGFR the distinct role of other HER receptor members, such as HER2 and HER3, in modulating mutant KRAS-driven tumorigenesis is not known. We therefore examined the collective contribution of EGFR, HER2 and HER3 in the molecular mechanism underlying the pathogenesis of KRAS mutant lung cancer. We present divergent cellular mechanisms associated with RTK-dependent and RTK-independent cell lines and provide *in vitro* and *in vivo* evidence demonstrating the anti-tumor efficacy of targeting EGFR/HER in the RTK-dependent subset. Our model suggests that in a group of mutant KRAS lung cancers, EGFR is not the major upstream signaling activator, but that this role is also played by HER2 and HER3. Multi-targeting the HER receptors may thus have positive implications for the treatment of tumors that harbor these specific mutant KRAS isoforms.

## RESULTS

### Silencing oncogenic KRAS in KRAS-dependent NSCLC cells

**Figure 1:**
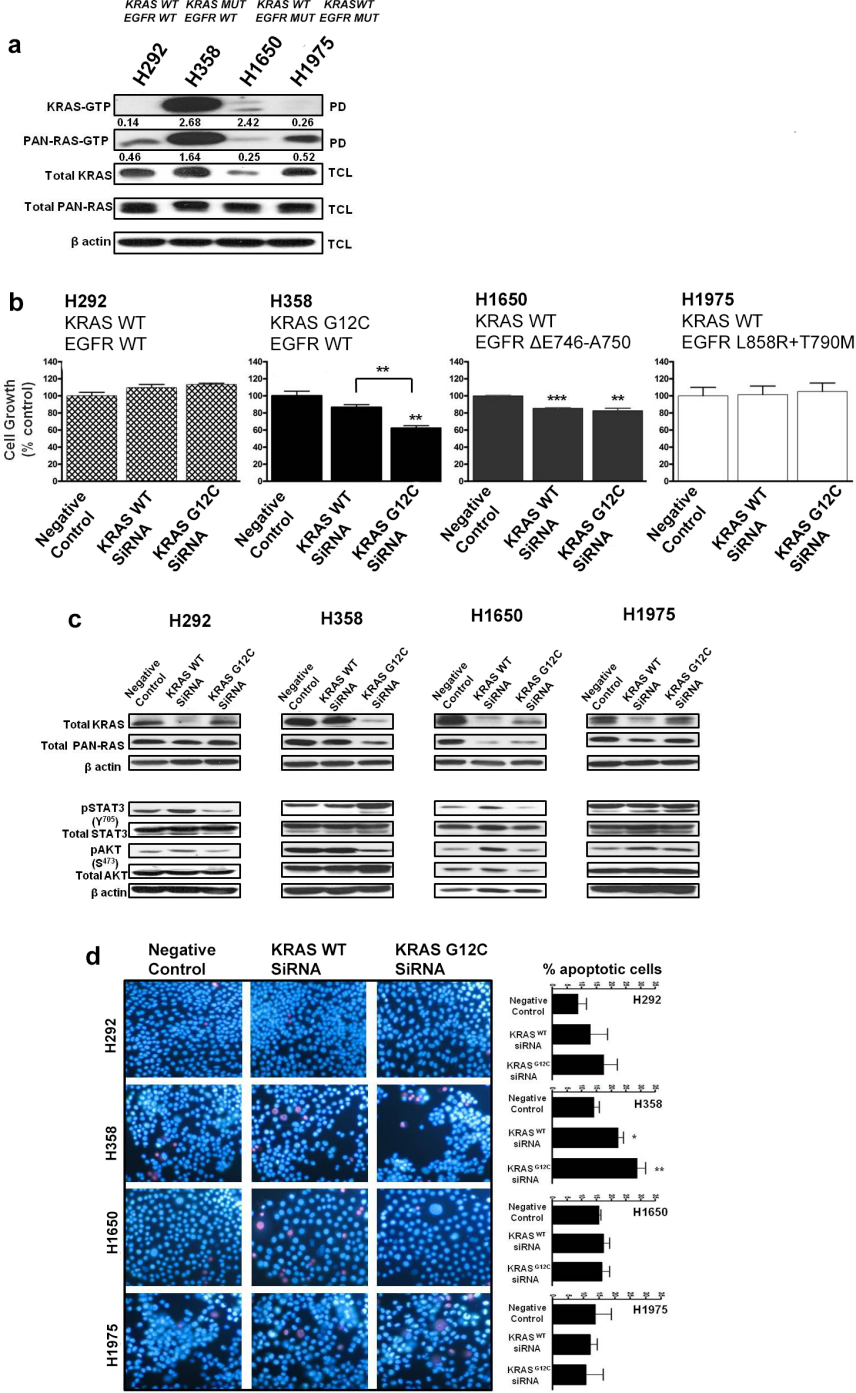
Silencing oncogenic KRAS in KRAS-addicted NSCLC cells **a.** Ras-GTP levels in NSCLC cells expressing mutant KRAS, mutant EGFR or their wild-type form were measured with a pull-down assay (PD). GTP-bound Ras, isolated from the PD and total cell lysate (TCL) subjected to immunoblot analysis are shown. Values represent normalized ratios of active RAS to total RAS levels, quantified by Image J analysis. **b.** NSCLC cells transiently transfected with wild-type KRAS or mutant KRAS (G12C) siRNA for 72 hrs were assessed for cell growth by MTS (values are representative of mean ± SEM of three independent experiments) and **c.** immunoblot analysis with the indicated antibodies. **d.** Cellular apoptosis was quantified by Hoechst 33342 (blue) and propidium iodide (red) double fluorescent chromatin staining on cell cultures 72 hrs post siRNA transfection. Representative images of two independent experiments from 3 to 5 randomly selected microscopic fields are shown (40× magnification). Also see Supplementary Figure S1.

Four human NSCLC cell lines with differing KRAS and EGFR mutational status, H292 (KRAS^wt^; EGFR^wt^), H358 (KRAS^G12C^; EGFR^wt^), H1650 (KRAS^wt^; EGFR^ΔE746-A750^) and H1975 (KRAS^wt^; EGFR^L858R + T790M^), were assessed for RAS-GTP activity by a Raf ‘pull down assay’ using the RAS-binding domain of Raf-1. H358 cells harboring oncogenic KRAS displayed elevated levels of active KRAS-GTP (isoform specific) and pan-RAS-GTP when compared to the other NSCLC cell lines (Fig. 1a). Interestingly, although H1650 cells express lower levels of total KRAS compared to the other cell lines, the normalized ratio of active KRAS-GTP to total KRAS was relatively high-a calculated ratio of 2.42 compared to a ratio of 2.62 for H358 cells (Fig. 1a). However, the overall KRAS-GTP signal observed in H1650 cells remains very low compared to H358 cells.

To also examine the respective roles of wild-type and mutant KRAS in the growth of H358 cells, siRNAs specific to wild-type KRAS and mutant KRAS G12C isoforms [17] were utilized in functional experiments. As shown in Fig. 1b, H358 cells exposed to mutant-specific KRAS siRNA displayed a ~40% reduction in cellular growth after 72 hrs (MTS assay), while a ~15% reduction was observed after wild-type KRAS siRNA treatment (Fig. 1b). Similar observations were seen with H23 (KRAS^G12C^; EGFR^wt^) cells (Fig. S1a). H1650 cells, carrying an activating EGFR mutation, demonstrated a ~15% significant reduction in cell growth after respective siRNA treatment with either wild-type or mutant KRAS (Fig. 1b). This observation could be as a result of the relatively enhanced levels of active KRAS seen in H1650 cells (Fig. 1a); possibly related to the absence of the PTEN phosphatase in this cell line [18]. No significant inhibitory effects were observed on the cellular growth of either H1975 cells carrying the EGFR^T790M^ resistance mutation or H292 control cells after similar treatments (Fig. 1b).

To determine the molecular changes associated with the decrease in cellular growth, we examined KRAS protein expression and effector signaling. A siRNA-mediated depletion of the wild-type KRAS isoform reduced the expression of KRAS in the control cell line as well as in the two EGFR mutant cell lines (Fig. 1c). In contrast, while knockdown of wild-type KRAS did not significantly reduce KRAS protein expression in H358 cells, mutant-specific knockdown potently and specifically reduced KRAS protein expression (Fig. 1c). Depletion of oncogenic KRAS impaired AKT phosphorylation in H358 cells, but resulted in a more robust induction of STAT3 phosphorylation at Tyr 705, compared to wild-type KRAS knockdown (Fig. 1c), indicating a feedback activation of STAT3. Similar results were also observed with the H23 cells harboring the same KRAS mutation (Fig. S1b). Our results show a modest reduction in phosphorylated STAT3 levels at Tyr 705 in H292 control cells with mutant KRAS G12C knockdown (Fig. 1c). The reduction of STAT3 could be the result of an miRNA effect [19], since sequence alignment of the mutant specific KRAS siRNA and EGFR reveals partial homologies, e.g. within the 3′ untranslated region of EGFR beginning at position 2098 (data not shown). In H1650 mutant EGFR cells, mutant KRAS knockdown also reduced KRAS protein levels but without a significant effect on the downstream signal transduction pathways (Fig. 1c). Again, this could be related to the relatively high levels of active KRAS seen in this cell line (Fig. 1a). The absence of effects on the downstream pathways was also observed in the H1975 cells.

To examine the effect of wild-type or mutant specific KRAS siRNA on the survival of the NSCLC cell lines, Hoechst 33342/Propidum Iodide (PI) double chromatin staining was performed to detect DNA condensation in cell cultures 72 hrs post-transfection. The number of apoptotic cells was calculated by microscopic examination of cells displaying blue nuclear fragmentation (Hoechst) and red nuclear fragmentation (PI), indicating the induction of early and late apoptosis. As shown in Fig. 1d, siRNA-mediated depletion of wild-type and mutant KRAS had an impact on the survival of H358 cells with a respective increase of 8.4 ± 0.2% (wild-type) and 14.8 ± 1.0% (mutant) in the number of apoptotic cells relative to control. In addition, H23 NSCLC cells treated under similar conditions displayed comparable results (Fig. S1c). No significant apoptotic effects were observed with the other NSCLC cell lines after similar siRNA treatment (Fig. 1d). Taken together, these results indicate that although knock down of KRAS G12C in dependent NSCLC cells causes growth inhibition and a modest induction of apoptosis, this effect may be attenuated by the upregulation of phosphorylated STAT3 and hence the increase of survival signals.

### EGFR/HER-dependent and EGFR/HER-independent isoforms of KRAS mutant NSCLC

**Figure 2:**
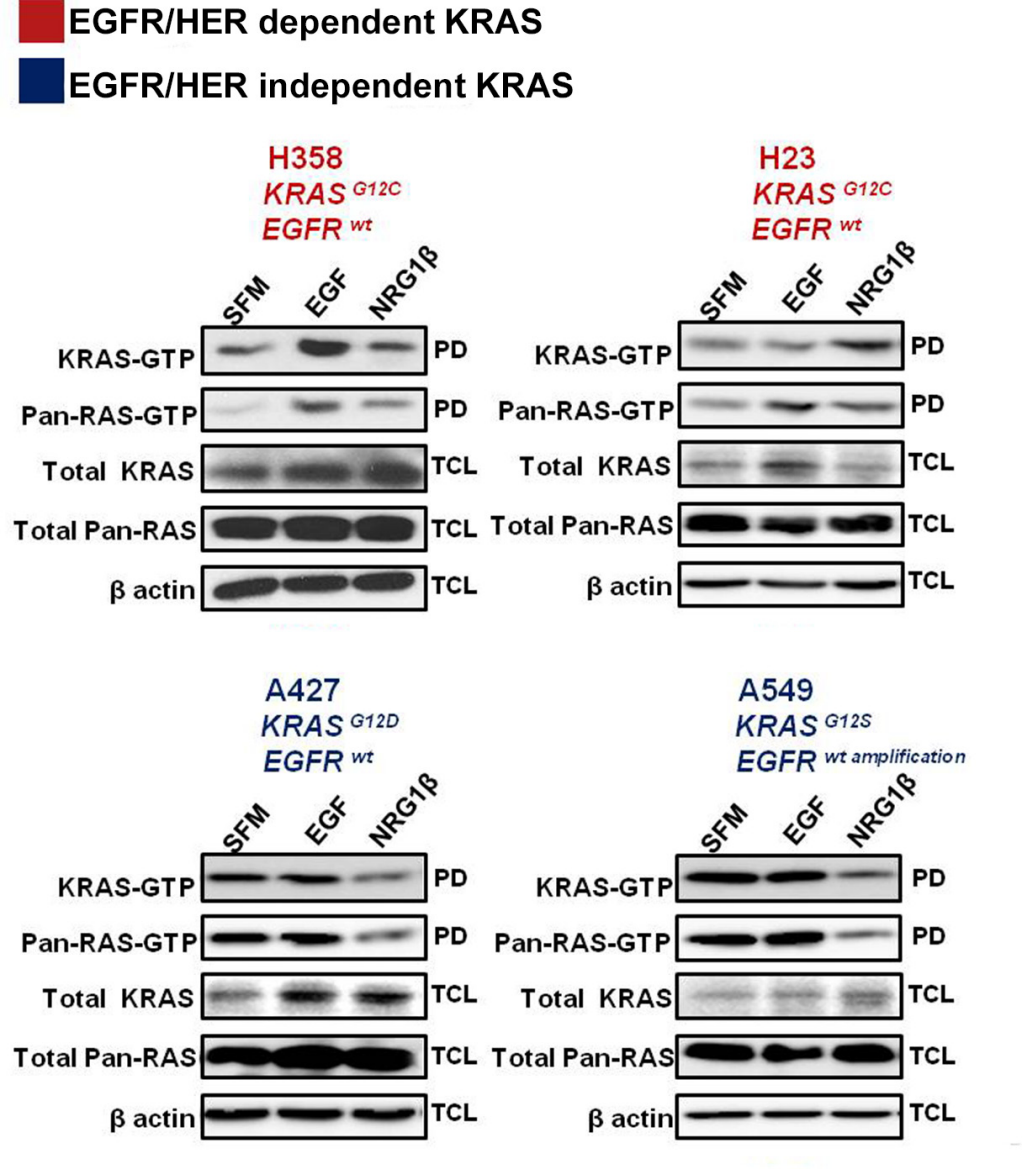
Effects of acute growth factor stimulation on RAS activity in mutant KRAS NSCLC cells NSCLC cells were serum starved for 16 hrs and stimulated for 10 minutes with the indicated ligands. Ras-GTPlevels were measured with a pull down (PD) assay and total cell lysates (TCL) were subjected to immunoblot analysis with the indicated antibodies.

KRAS is central to multiple signaling cascades and has been shown to induce growth factor-independence and constitutively activate downstream signaling effectors in its oncogenic form [13]. To further investigate whether oncogenic KRAS activity could be influenced by stimulation of EGF or other HER-family related ligands, a panel of mutant KRAS NSCLC cells - H358 (KRAS^G12C^; EGFR^wt^), H23 (KRAS^G12C^; EGFR^wt^), A427 (KRAS^G12D^; EGFR^wt^) and A549 (KRAS^G12S^; EGFR^wt amplification^) - were serum starved overnight and then stimulated with epidermal growth factor (EGF) or neuregulin 1β (NRG1β) for ten minutes. As shown in Fig. 2, acute stimulation of H358 cells with EGF, strongly enhanced KRAS-GTP and pan-RAS-GTP loading, while treatment with NRG1β, the ligand for HER3, mostly enhanced pan-RAS GTP levels. In contrast, although EGF stimulation enhanced pan-RAS-GTP levels in H23 cells bearing the same KRAS^G12C^ mutation as H358 cells, it was NRG1β stimulation that mostly enhanced active KRAS levels (Fig. 2). These data indicate that H358 and H23 cells are dependent on extracellular growth signals from the EGFR or HER2/HER3 for enhanced KRAS GTP-loading. Examination of active RAS levels in the two other KRAS mutant isoforms (A427 and A549) did not reveal an increase in either KRAS or pan-RAS GTP loading upon EGF stimulation (Fig. 2), pointing to a EGFR-independent phenotype in these cells. To the contrary, NRG1β stimulation even decreased their active KRAS and pan-RAS levels further indicating that A427 and A549 cells are differentially influenced by upstream stimulation of the EGFR/HER receptors.

### Silencing the EGFR/HER in KRAS mutant NSCLC cells

**Figure 3:**
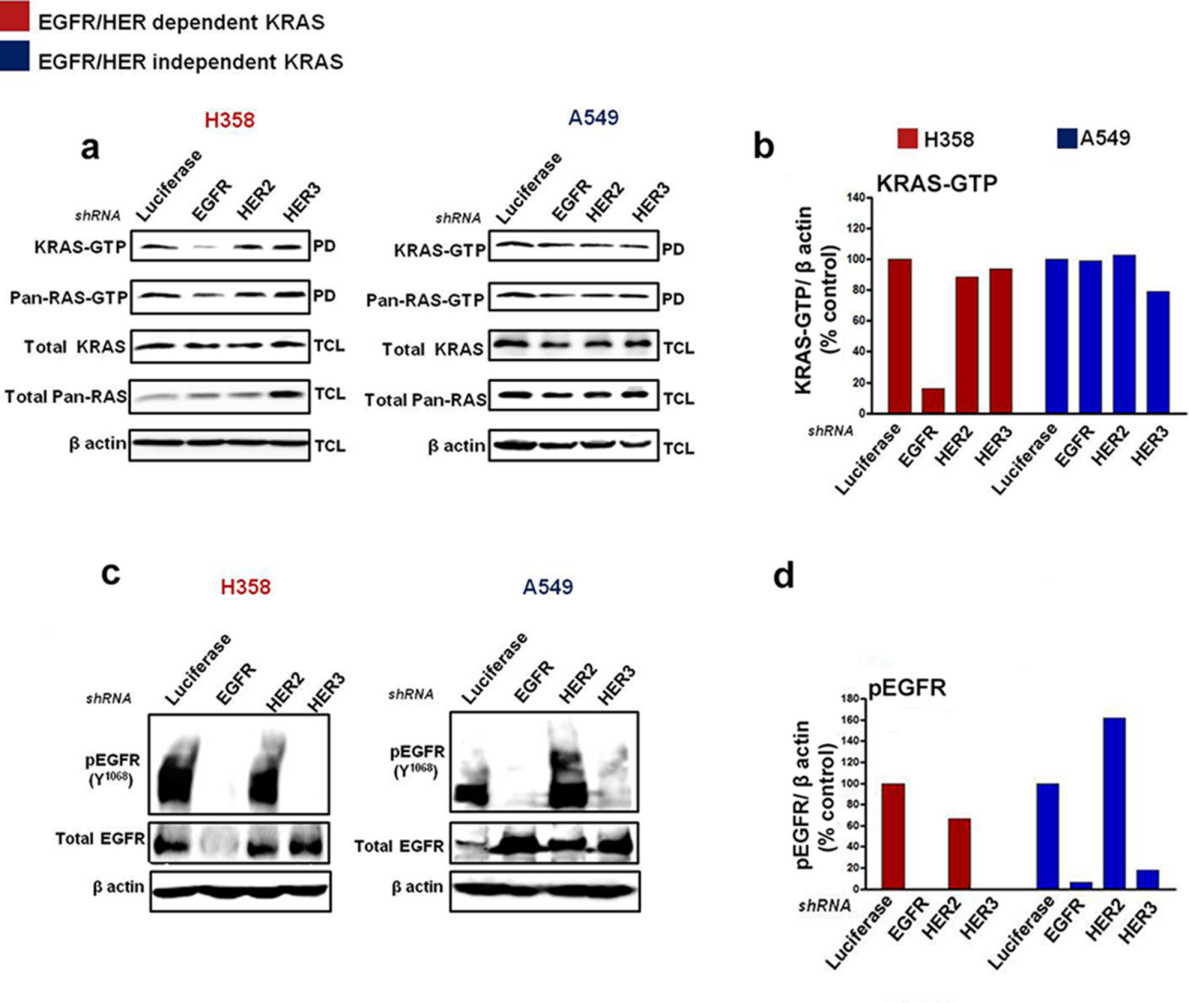
Silencing EGFR/HER in mutant KRAS NSCLC cells Cells were transfected with shRNA targeting Luciferase, EGFR, HER2 and HER3 and enriched with puromycin (see Materials and Methods). Ras-GTP levels were measured with a pull down (PD) assay and total cell lysates (TCL) were subjected to immunoblot analysis with the indicated antibodies **a, c. b, d.** Values of KRAS-GTP and phosphorylated EGFR levels from the immunoblot analyses in (a, c) are shown in histograms. Values are normalized relative to Luciferase shRNA treatment as quantified by Image J analysis. Also see Supplementary Figure S2.

To evaluate how KRAS-driven NSCLC cells are regulated by the HER receptors, we performed short hairpin (shRNA) -mediated knockdown of EGFR, HER2 and HER3 on two candidate cell lines from the EGFR/HER dependent (H358) and EGFR/HER independent (A549) groups (as evaluated in Fig. 2). The cells were transfected with the shRNA plasmids, enriched in medium containing puromycin; followed by assessments of active KRAS-GTP and EGFR/HER levels. shRNA-mediated knockdown of EGFR in H358 cells potently reduced, by approximately five-fold, active KRAS-GTP levels compared to shRNA targeting the luciferase control gene, as well as HER2 and HER3 (Fig. 3a & 3b). For A549 cells, treatment with the HER shRNAs did not induce a significant depletion in KRAS-GTP levels (Fig. 3a & 3b). Combined knockdown of EGFR and HER2 potently reduced KRAS-GTP levels in H358 cells, while similar shRNA treatment enhanced KRAS-GTP levels in A549 cells (Fig. S2a).

EGFR and HER3 shRNA-mediated knockdown in H358 cells potently reduced phosphorylated and total levels of EGFR compared to control (Fig. 3c & 3d). Importantly, combined knockdown of EGFR and HER2 also produced a marked decrease in levels of phosphorylated and total EGFR in H358 cells (Fig. S2b). Although a potent reduction in EGFR phosphorylation was observed in A549 cells after EGFR or HER3 shRNA treatment, a corresponding increase in total EGFR levels was also observed in this cell line (Fig. 3c & 3d). Furthermore, treatment with HER2 shRNA in A549 increased both phosphorylated and total levels of EGFR (Fig. 3c & 3d), while the combination of EGFR and HER2 shRNA also correlated with an increase in EGFR expression levels (Fig. S2b). The reason behind these observations remain unclear, but could be linked to an unknown feedback mechanism between the HER receptors that render mutant KRAS independent of EGFR/HER regulation in A549 cells.

### Effect of combined HER inhibition in KRAS and EGFR mutant NSCLC cell lines

Oncogenic KRAS and its related signaling effectors are thus readily activated by upstream HER stimulation in certain KRAS^G12C^ NSCLC cells (see above and Fig. 2). We subsequently investigated the effect of targeting oncogenic KRAS activity indirectly, by combined inhibition of the HER family of receptors with the EGFR small molecule tyrosine kinase inhibitor erlotinib, and with the HER2-specific monoclonal antibody pertuzumab. Pertuzumab prevents HER2 from dimerizing with its other HER partners thereby blocking signaling [20]. The same panel of KRAS mutant NSCLC cells (H358, H23, A427 and A549) previously tested (Fig. 2), was compared to mutant EGFR NSCLC cells (H1650 and H1975) that have a wild-type KRAS status.

**Figure 4:**
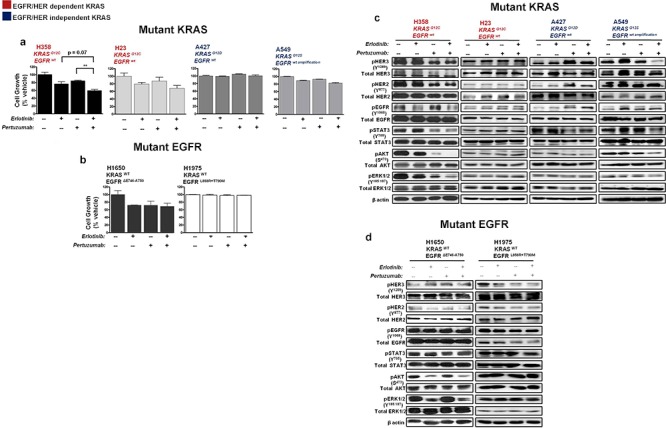
Effect of erlotinib and pertuzumab combination on growth and EGFR/HER signalling in KRAS and EGFR mutant NSCLC cells Cells were treated with erlotinib (100 nM), pertuzumab (25 μg/ml) or their combination for five days. **a, b.** Growth inhibition was determined by the SRB *in vitro* toxicology assay. Data are expressed relative to control and are representative of mean ± SEM of two to three independent experiments **c, d.** Total cell lysates were subjected to immunoblot analysis with the indicated antibodies. Also see summary table (Table S1).

The NSCLC cells were treated for five days with erlotinib (100 nM), pertuzumab (25 μg/ml), their combination, or vehicle control and biological effects were examined. All of the NSCLC cell lines display resistance to clinical achievable doses of single-agent erlotinib or pertuzumab (data not shown). As shown in Fig. 4a, combined treatment significantly reduced *in vitro* cellular growth of H358 cells compared to single treatment conditions and a more modest reduction in the growth of H23 and A549 cells. In contrast, combination treatment yielded no growth inhibition in A427 cells (Fig. 4a).

Combination treatment in H358 cells reduced EGFR and HER2/3 phosphorylation resulting in the suppression of downstream effectors: STAT3, AKT and ERK1/2 (Fig. 4c; also see summary table - Table S1). In H23 cells, active levels of EGFR/HER and downstream signaling effectors were unaffected (Fig. 4c). In A549 cells, erlotinib had a paradoxical effect of stimulating the phosphorylation status of EGFR and HER3, while the combination with pertuzumab caused only some inhibition of active HER3 levels, but not of EGFR or HER2 (Fig. 4c; Table S1). This further indicates that an unknown feedback mechanism affecting EGFR/HER2 could be active in these KRAS mutant cells bearing an EGFR amplified status or that the dose of erlotinib employed was insufficient to block the overexpressed EGFR protein. In the same vein, an increase in active levels of all three HERs was observed in A427 cells upon pertuzumab treatment, while combination treatment yielded no significant effects on active levels of HER and downstream effector proteins AKT and ERK1/2 (Fig. 4c; Table S1).

The dual effect of erlotinib and pertuzumab on growth and EGFR/HER activity of H1650 and H1975 EGFR mutant NSCLC cells was less evident. No pronounced effects were observed in comparison to single treatment conditions (Fig. 4b and 4d).

### Combined HER inhibition promotes anti-tumor efficacy in EGFR/HER-dependent KRAS mutant NSCLC

**Figure 5:**
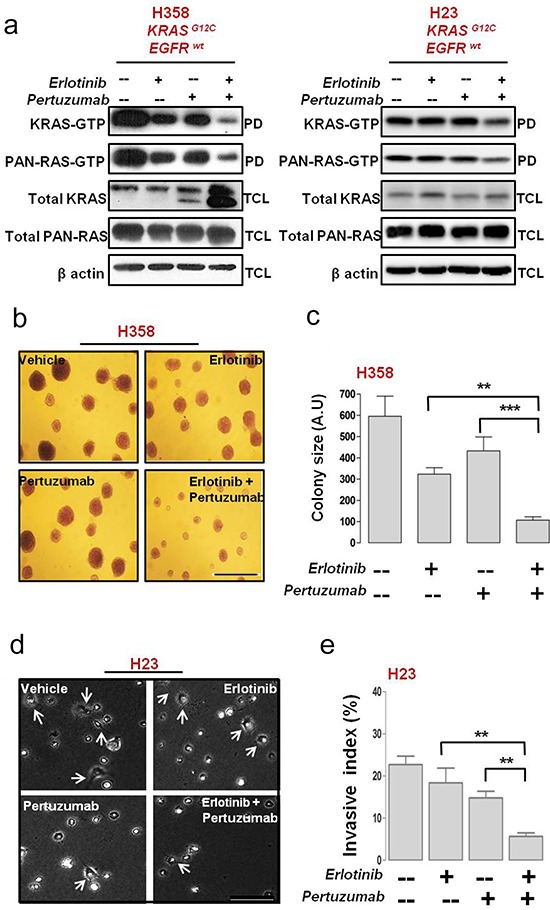
Anti-tumor efficacy of erlotinib and pertuzumab combination in EGFR/HER-dependent KRAS mutant NSCLC cells H358 and H23 NSCLC cells were treated with erlotinb (100 nM), pertuzumab (25 μg/ml) or their combination for five days. **a.** Active bound Ras-GTP levels in mutant KRAS H358 cells were measured with a pull-down (PD) assay, and total cell lysate (TCL) subjected to immunoblot analysis are shown. **b.** H358 cells were cultured for 21 days on a collagen type I gel in the presence of the indicated inhibitors. Colonies were fixed and stained with crystal violet and bright-field images are representative of 20× magnification (Bar, 100 μm) **c.** Quantification of colonies formed by H358 cells after inhibitor treatment. **d.** Phase contrast images of H23 cells cultured on a collagen type I gel for 48 hrs in the presence of the indicated inhibitors. Arrows indicate invasive extensions and images are representative of 40× magnification (Bar, 50 μM). **e.** Invasive index quantification of H23 cells after inhibitor treatment. Values from (c) and (e) are representative of mean ± SEM.

Combined erlotinib and pertuzumab treatment severely reduced KRAS and pan-RAS GTP loading in H358 cells, significantly more than either treatment alone (Fig. 5a; H358). Interestingly, KRAS protein expression was strongly upregulated in H358 cells (doublet band in Fig. 5a; H358) upon combined treatment. In H23 cells, there was also a reduction in both KRAS and pan-RAS GTP levels compared to single treatment conditions (Fig. 5a).

We further examined the impact of combined HER inhibition on the invasive capabilities of H358 and H23 cells, by culturing the cells on a type I collagen substrate in the presence of the inhibitors or their combination. Of note, H358 cells typically form colonies on top of collagen whereas H23 cells attach and form invasive extensions inside the substrate. Combining erlotinib and pertuzumab reduced the tendency of H358 cells to form colonies on a type I collagen matrix (Fig. 5b); with a six-fold reduction in colony size observed after combined treatment (Fig. 5c). The invasive growth of H23 cells was attenuated after 48 hrs post combination treatment, as depicted by the reduced number of invasive extensions (Fig. 5d). Quantification demonstrated that combined treatment exerted a 17.02 ± 1.2% (*p* < 0.01) or a four-fold decrease in the invasive capacity of H23 cells compared to vehicle control (Fig. 5e).

**Figure 6:**
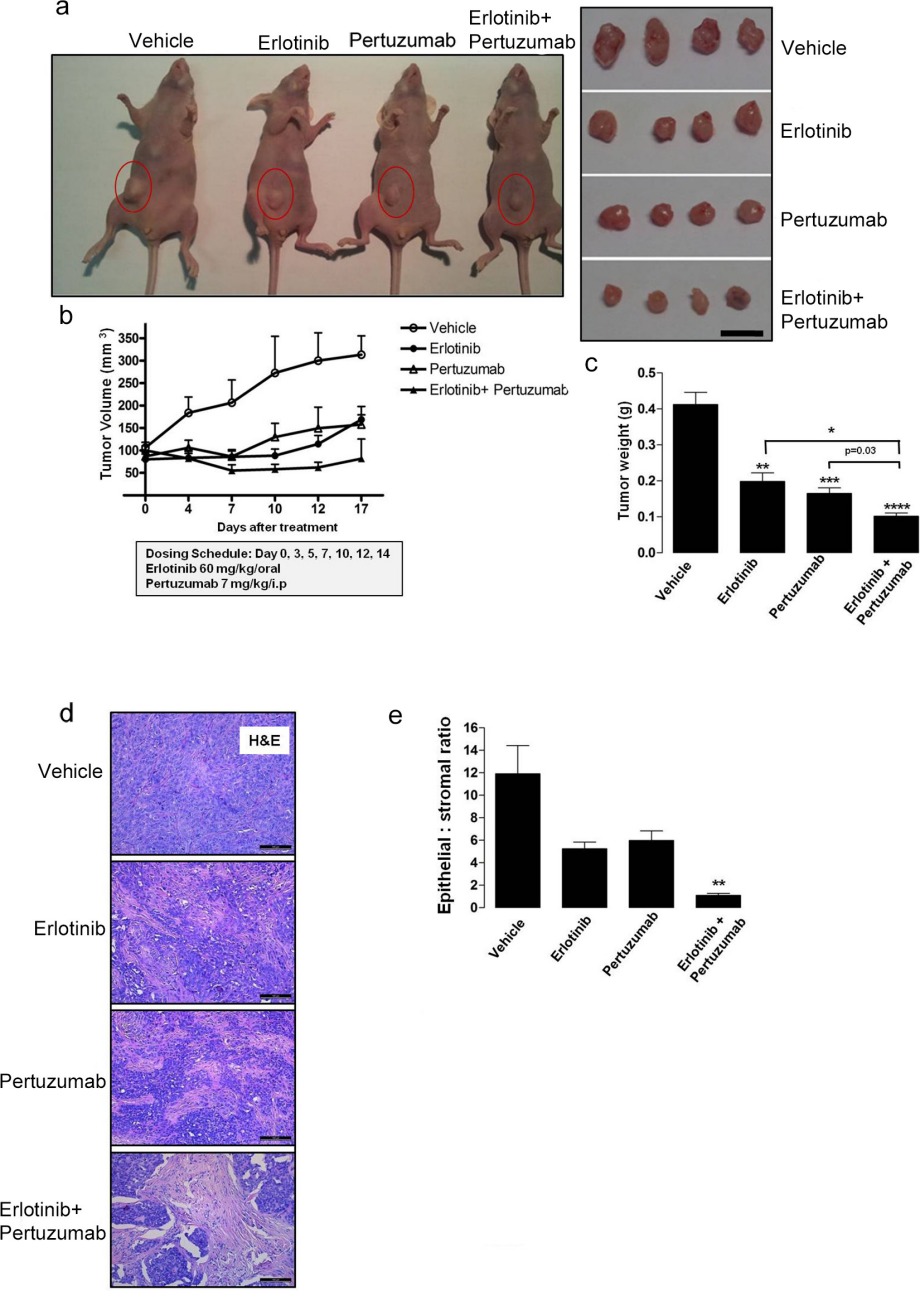
Anti-tumor efficacy of sequential erlotinib/pertuzumab treatment in KRAS mutant xenografts KRAS mutant H358 cells were implanted subcutaneously into nude mice until tumors reached a volume of approximately 100 mm^3^. Xenografts (*n* = 8) were randomized and received vehicle, 60 mg/kg erlotinib, 7 mg/kg pertuzumab or their combination three days a week for 17 days. **a.** Representative images of tumor bearing mice and resected tumors from respective treatment groups. Scale bar, 10 mm **b.** Change in tumor volume of individual treatment groups. **c.** Comparison of tumor weight from respective treatment groups. **d.** Representative H&E stained mouse tumor sections from respective treatment groups. Scale bar, 100 μM **e.** Quantification of epithelial to stromal ratio of H&E stained tumor sections from individual treatment groups. Values from (b), (c) and (e) are representative of mean ± SEM. Also see Supplementary Figure S3.

Finally, to evaluate the *in vivo* anti-tumor efficacy of combined erlotinib and pertuzumab treatment in NSCLC-derived mutant KRAS^G12C^, we examined a mouse model bearing xenografts of H358 cells. Tumor bearing mice were randomized into individual groups (*n* = 8) and treated with either vehicle, single or sequential combination treatment ([erlotinib 60 mg/kg/oral; pertuzumab 7 mg/kg/i.p.] 3x weekly) for 17 days. As shown in Figures 6a, 6b and 6c, sequential combination treatment reduced tumor volume and tumor weight after 17 days. In addition, a 50 ± 12.9% reduction in tumor volume was observed in the combination treatment group already within seven days of treatment (Fig. 6b). Histological analysis of the xenografted tissue revealed a lower amount of the epithelial component and higher amount of stroma in the combined treatment group compared to the other groups (Fig. 6d). This observation further demonstrates reduced tumor cell growth in this dual treatment cohort. In addition, some tumor sections from the combination treatment group exhibited pyknotic nuclei, suggesting the induction of apoptosis. This observation was not made in analyzed tumor sections from the other treatment groups. Moreover, quantification of the epithelial to stromal ratio confirmed that this ratio was strongly decreased in the combined treatment group, approximately 12 fold when compared to vehicle control, and approximately 5 and 6 fold when compared to erlotinib and pertuzumab treatment groups respectively (Fig. 6e). Ki67 cellular proliferation immunostaining was also performed on the tumor sections, but did not display any distinct variation across the four groups (data not shown). The Ki67 index was low in all treatment groups. The mouse tumor sections were further analyzed for the presence of DNA fragmentation and late apoptosis using a TUNEL-based assay. The analysis revealed a modest 1.5 to 2-fold increase in TUNEL positive cells in the combined treatment group compared to the other respective groups (Fig. S3c).

To determine the status of active EGFR/HER and active KRAS/pan-RAS levels across all treatment groups, we performed both immunoblot analysis and a Raf ‘pull down assay’ on cell lysates extracted from crude tumor tissue. Comparison of active EGFR/HER levels in the individual treatment groups demonstrated that combined treatment potentiated a decrease in phosphorylated EGFR at activating residues (Tyr 1068 and Tyr 1173) as well as a corresponding decrease at the activating Tyr 1248 residue of HER2 (Fig. S3a). Importantly, levels of active EGFR/HER were higher in the single treatment groups compared to vehicle control (Fig. S3a). As shown in Fig. S3b, active KRAS levels were severely reduced in the combination treatment group, consistent with our results obtained *in vitro*. Surprisingly, active pan-RAS levels remained unaffected across all groups suggesting that EGFR/HER inhibition is specific to the KRAS-addicted phenotype of H358 cells.

## DISCUSSION

In the current study, we investigated upstream events that may influence the mutant KRAS signaling pathway in NSCLC. We found that EGF or NRG1β stimulation can strongly enhance the amount of active RAS in NSCLC cells harboring oncogenic KRAS^G12C^. In accordance with our findings, a recent report demonstrates that oncogenic KRAS requires EGF stimulation for enhanced activity in pancreatic cancer cells: EGF induced a prolonged activity of oncogenic KRAS, whereas only transient effects are seen in cells with wild type KRAS [15]. Surprisingly, while EGF stimulation had no effect in increasing the levels of KRAS-GTP in some other examined mutant isoforms of KRAS, NRG1β treatment reduced active levels of KRAS in these cell lines. The explanation for this remains unclear, but this observation could be in accordance with previous data that indicate a possible tumor suppressor function of NRG1 [21]. Our observations disagree with previous reports which describe oncogenic KRAS as solely constitutively active and independent from upstream activation by EGFR or other RTKs [13, 22–25]. Although this is the case in some RAS mutant tumor types, including some of the NSCLC tested here, our work indicates that some KRAS mutant cancers retain sensitivity to upstream stimuli emanating from extracellular signaling in particular via the HER pathway. These data therefore indicate that mutant KRAS isoforms can be classified into two groups: (1) EGFR/HER/RTK -dependent or (2) EGFR/HER/RTK-independent. To our knowledge, this is the first report that clearly delineates HER dependent or independent groups in the context of NSCLC-derived mutant KRAS.

We found that silencing oncogenic KRAS in EGFR/HER-dependent NSCLC cells reduced cellular growth, and also induced a modest apoptotic signal. These findings are consistent with previous reports on pancreatic cancer cell models [17, 26]. While the depletion of KRAS expression by mutant-specific siRNA was accompanied by a reduction in AKT phosphorylation levels in the EGFR/HER-dependent subset, it was also followed by robust activation of STAT3. These results suggest a feedback loop via STAT3 that re-establishes oncogenic signaling, compensating for the loss of AKT survival signals. Our results are compatible with findings of Sunaga et al. [27], who showed that there is a feedback loop involving autocrine secretion of TGFβ, and subsequent phosphorylation of EGFR and STAT3.

The sole silencing of oncogenic KRAS may thus not be an effective therapeutic strategy in KRAS-addicted cancers, since upstream events and feedback loops are likely to attenuate or annul the effects of the therapeutic intervention. To further determine how mutant KRAS is regulated by EGFR and other HER members, we investigated shRNA-mediated knockdown of EGFR, HER2 and HER3 in cell lines classified as EGFR/HER-dependent or independent. Contrary to the current model of a constitutively active mutant KRAS, we found that knockdown of EGFR, successfully reduced active KRAS-GTP levels in the cell line representing the EGFR/HER-dependent subset. Importantly, we also show that knockdown of the HER receptors potently suppressed phosphorylated and total levels of their proteins in the dependent subset. In contrast, while shRNA-mediated knockdown of EGFR/HER had no significant effects in reducing KRAS-GTP levels in the A549 cell line representing the EGFR/HER independent subset, knockdown of HER2 led to the robust upregulation of pEGFR and total EGFR in this cell line. We do not have a mechanistic explanation for this observation but our data suggest a feedback between the HER receptors that makes the mutant KRAS independent from EGFR/HER regulation.

We and others have previously shown that NSCLC cells carrying an oncogenic KRAS mutation exhibit primary resistance to anti-EGFR therapy with small molecule inhibitors such as erlotinib, and propose that these KRAS-addicted cells subvert upstream signals emanating from EGFR [28]. To confront these earlier findings with our current data, we inhibited EGFR, HER2 and HER3 with a combination of erlotinib and pertuzumab leading to the suppression of *in vitro* growth and the inhibition of receptor signaling and downstream signaling effectors in the dependent subset. In contrast we found a paradoxical increase in phosphorylated EGFR levels after dual treatment in KRAS mutant A549 cells that harbor a wild-type EGFR amplification. Consistent with these molecular observations, we also did not observe a significant suppression of *in vitro* cellular growth in these cells. While amplification of the EGFR has been reported to predict response to EGFR TKIs [29], its concomitance with oncogenic KRAS seemingly acts as a driver of resistance. KRAS mutant A427 (EGFR/HER-independent) cells were highly unresponsive to the drug combination treatment and also rather showed an increase in active HER receptor levels compared to vehicle control. These cell lines thus represent KRAS mutant NSCLC that are EGFR/HER independent, at least from a therapeutic perspective.

We confirmed the anti-tumor effects of a combined HER blockade, in mouse xenografts specifically affecting the epithelial component of the xenografts. Consistent with our findings, erlotinib combined with pertuzumab has been shown to surpass single therapy in inhibiting tumor activity in a NSCLC xenograft model with high EGFR protein expression (QG-56 cells) [30]. Moreover, preliminary observations from a small phase Ib clinical trial (*n* = 15) have revealed that erlotinib and pertuzumab combination therapy is well tolerated and could be suitable for further clinical testing in phase II [31]. Further exploration in larger cohorts should include the assessment of biomarkers such as KRAS mutation status and preferably be performed in patients that are early enough in their disease course and have a sufficient life expectancy to allow for a correct evaluation of treatment responses and outcome.

Possibly in line with our observations, Metro et al. recently found a differential clinical outcome for specific KRAS oncogene substitutions when treated with single agent erlotinib only [32]. They concluded that although EGFR-TKI intervention may not be effective in the mutant KRAS/wild-type EGFR NSCLC cohort as a whole, it may still beneficial to patients harboring specific KRAS mutations. The clinical experiment was not ideal to test the efficacy of targeted therapies as this was in end-stage NSCLC patients with a very limited life expectancy. Nevertheless one of two responders was in a patient harboring a KRAS^G12C^ mutation in their tumor similar to our EGFR/HER dependent cells. The other responder was in a KRAS^G12A^ bearing tumor, not represented in the panel examined in our study.

In conclusion, our results point to the important role of the EGFR/HER interaction in KRAS activation and strongly suggest that in a subset of NSCLCs, mutant KRAS is not an independent and constitutive signal transduction molecule. A multi-targeted strategy that can abrogate the EGFR/HER/KRAS interaction may thus have a therapeutic perspective. This strategy should thus be explored in a clinical setting using molecular characterized NSCLCs and possibly in other cancer types as well.

## MATERIALS AND METHODS

### Cell lines and reagents

The human NSCLC cell lines H292 (CRL-1848™), H358 (CRL-5807™), H23 (CRL-5800™), A427 (HTB-53™), H1650 (CRL-5883™) and H1975 (CRL-5908™) were obtained from the American Type Culture Collection, while the A549 NSCLC cell line was obtained from Sigma. All cell lines were genotyped by STR analysis. The inhibitors, erlotinib and pertuzumab were provided by Roche.

### Western blot analysis and antibodies

To analyze tyrosine phosphorylation of the HER receptors, their downstream signalling targets and KRAS signalling effectors, NSCLC cells were seeded in clear-bottomed 24 well plates at a density of 2 × 10^5^. After 24 hrs, all cells received fresh media with or without inhibitor or their combination and were further incubated for an additional 48 hrs; receiving fresh media and inhibitor daily. Cells were lysed in a Tris-buffer [25 mmol/L Tris-HCL (pH 7.4), 150 mmol/L NaCl, 1% Triton-x, 5 ug/ml leupeptin] containing a protease and phosphatase inhibitor cocktail (*Sigma*). Lysates were cleared by centrifugation and protein concentration was determined by the Bradford protein assay kit (*Bio-Rad*) and equivalent amount of protein were loaded on an 8.5% resolving acrylamide gel and blotted on a polyvinylidene fluoride membrane (PVDF). The membrane was then subjected to an immunodetection procedure using the indicated antibodies: phospho-EGFR (Tyr 1086) phospho-HER2 (Tyr 887), phospho-HER3 (Tyr 1289), phosphor-STAT3 (Tyr 705), EGFR, HER2, HER3, ERK1/2, AKT/PKB, STAT3 from Cell Signaling, phospho-ERK1/2 (Tyr185/187), phospho-AKT/PKB (Ser 473) from Invitrogen, and β-actin from Sigma-Aldrich. Horseradish peroxidase (HRP) - conjugated secondary antibodies (*GE Healthcare; Cell Signalling*) and a chemoluminescent detection kit (*Perkin-Elmer*) were used to detect the indicated proteins.

For RAS-GTP pull down assays, cells were cultured, collected and lysed. RAS-GTP was pulled down using the RAS-binding domain (RBD) of Raf-1 from the RAS activation kit (*Millipore*) according to the manufacturer's instructions with modifications. An isoform-specific KRAS antibody and a pan-RAS antibody were used for protein detection by western blot.

### Drug inhibition

The effect of drug treatment on cellular growth was assessed by the MTT based Sulforhodamine B (SRB) *in vitro* toxicology assay (*Sigma*). Briefly, cells were seeded in clear-bottomed 96-well plates at a density of 2 × 10^3^ cells/well in triplicates and treated after 24 hrs with concentrations of the indicated drugs or their combination. After the indicated time periods, the numbers of viable cells were fixed (trichloroacetic acid) and then stained with SRB. The wells were measured at an absorbance of 490 nm using a 96-well microplate reader (*Labsystems*) according to the manufacturer's instructions. All drug treatment conditions were compared to the vehicle control.

### RNA interference

Wild-type and mutant-specific KRAS siRNA were synthesized (*Eurogentec*) according to a previously described sequence [17]. The following oligonucleotide sequences were used for KRAS, 5′-GUUGGAGCUGGUGGCGUAG-3′ and for KRAS G12C, 5′-GUUGGAGCUUGUGGCGUA-3′. Briefly, cells were seeded in clear-bottomed 24-well or 96-well plates at a density of 0.7 to 1.5 × 10^5^ cells/well (24 well plate) or 2 to 8 × 10^3^ cells/well (96 well plate) respectively. 24 hrs later, cells were transfected with 100 nmol/L of siRNA (or 50 nmol/L for their combinations) using Lipofectamine 2000 (*Invitrogen*) according to the manufacturer's instructions. Transfected cells were cultured at 37°C for the indicated time periods and collected and lysed for immunoblot analysis with the indicated antibodies or analyzed for cell growth by MTS analysis (*Promega*).

ShRNA constructs were based on the pKAR1/Pur plasmid (Addgene plasmid # 23105); a gift from Randy Poon [33]. Specific shRNA constructs were created by cloning the following pairs of oligonucleotides into the *BbsI* and *XbaI* sites of pKAR1/Pur: EGFR: 5′-GAGGAAAUAUGUACUACGA-3′, HER2:5′-GGACGAAUUCUGCACAAUG-3′, HER3:5′GCAGUGGAUUCGAGAAGUG-3′ and Luciferase: 5′ GCCAT TCTATCCTCTAGAGGATG 3′. The shRNA-expressing plasmids were transfected into the NSCLC cells using Lipofectamine 2000 as described. After 48 hrs, medium was replaced with fresh medium containing 0.5 μg/ml puromycin to enrich the transfected cells. Puromycin containing medium was removed after 36 hrs and cells were grown in standard growth medium for 3 days. Cell lysates were collected and analyzed for RAS-GTP pull down and immunoblot analysis using the indicated antibodies.

### Hoechst 3342/propidium iodide staining

The effects of wild-type or mutant (G12C) KRAS siRNA treatment on apoptosis and nuclear morphology in the NSCLC cells was assessed by Hoechst 33342 and propidium iodide (PI) double fluorescent chromatin staining as described [28].

### Collagen assay

The Collagen assay was performed as described [34]. Briefly, cells (1 × 10^5^) were seeded on a collagen type I gel with or without treatment of the indicated inhibitors or their combination for the indicated time period. Collagen matrices were imaged under a phase-contrast light microscope and the invasive index was determined by manual counting of the number of invading cells (invasive extensions) and non-invading cells present in 5–10 randomly selected microscopic fields.

For the 21 day collagen assay, collagen matrices were fixed in 3% paraformaldehyde and stained with a 0.001% crystal violet solution followed by imaging under a light microscope with 10–15 randomly selected 200× microscopic fields in each well. Quantification of colony size was performed by counting the number of pixels by computerized Photoshop CS6 analysis (*Adobe*).

### Xenograft studies

Approximately 4.5 × 10^6^ cultured H358 NSCLC cells were subcutaneously injected into the flank of female athymic Swiss nu/nu mice (6 weeks old). Tumor-bearing mice were randomized into four experimental groups at 8 mice per group when the mean tumor volume was approximately 80 to 100 mm^3^. Randomized groups were treated with erlotinib by oral gavage at a dose of 60 mg/kg/every 3 days in 0.5% methylcellulose (*Sigma*). Pertuzumab was administered intraperitoneally (i.p.) at a dose of 7 mg/kg/every 3 days. In combination studies, groups of tumor-bearing mice received both erlotinib (60 mg/kg/every 3 days, orally) and pertuzumab (3 mg/every 3 days, i.p). Control animals were given both vehicles. Tumors were measured with vernier calipers and tumor volume estimated from 2-dimensional measurements using a prolate ellipsoid equation [Tumor Volume mm^3^ = (length × width^2^) × 0.5]. All studies were conducted after review by the Universiteit Gent Animal Care and Use Ethical Committee, in accordance with institutional guidelines.

### Immunohistochemistry

Xenografted tumor tissue was excised 21 days post dose, fixed in 10% formalin and paraffin-embedded. Hematoxylin and eosin (H&E) assessment for general morphology was subsequently performed.

### Statistical analysis

Results are representative of three independent experiments unless stated otherwise. Values are presented as the mean ± standard error of mean (SEM). The unpaired two tailed *t*-test was utilized to compare the means of two groups and the Chi-Square test was utilized for group comparison in the single cell collagen invasion model. Statistical significance is reported as follows: *P < 0.05, **P < 0.01, ***P < 0.001 and ****P < 0.0001.

## SUPPLEMENTARY DATA


