# Supplementary material for: Combined targeting of EGFR/HER promotes anti-tumor efficacy in subsets of KRAS mutant lung cancer resistant to single EGFR blockade
Source: Oncotarget. 2015 May 15;6(24):20132–44. doi: 10.18632/oncotarget.3853 (PMC4652993; doi:10.18632/oncotarget.3853)
Supplement: Supplementary file 1 [file oncotarget-06-20132-s001.pdf]

SUPPLEMENTARY DATA

Western blot analysis

To analyze tyrosine phosphorylation of the HER receptors, their downstream signalling targets and KRAS signalling effectors, NSCLC cells or crude tumor tissue were lysed in a Tris- buffer [25 mmol/L Tris-HCL (pH 7.4), 150 mmol/L NaCl, 1% Triton-x, 5 ug/ml leupeptin] containing a protease and phosphatase inhibitor cocktail (*Sigma*). Lysates were cleared by centrifugation and protein concentration was determined by the Bradford protein assay kit (*Bio-Rad*) and equivalent amount of protein were loaded on an 8.5% resolving acrylamide gel and blotted on a polyvinylidene fluoride membrane (PVDF). The membrane was then subjected to an immunodetection procedure using the indicated primary antibodies. Horseradish peroxidase (HRP) - conjugated secondary antibodies (*GE Healthcare; Cell Signalling*) and a chemoluminescent detection kit (*Perkin-Elmer*) were used to detect the indicated proteins.

For RAS-GTP pull down assays, NSCLC cells or crude tumor tissue were lysed and collected. RAS-GTP was pulled down using the RAS-binding domain (RBD) of Raf-1 from the RAS activation kit (*Millipore*) according to the manufacturer's instructions with modifications. An isoform-specific KRAS antibody and a pan-RAS antibody were used for protein detection by western blot.

RNA interference

Wild-type and mutant-specific KRAS siRNA were synthesized (*Eurogentec*) according to a previously described sequence [1]. The following oligonucleotide sequences were used for KRAS, 5′-GUUGGAGCUGGUGGCGUAG-3′ and for KRAS G12C, 5′-GUUGGAGCUUGUGGCGUA-3′. Briefly, H292 cells were seeded in clear-bottomed 24-well or 96-well plates at a density of 0.7 to 1.5 × 10^5 cells/well (24 well plate) or 2 to 8 × 10^3 cells/well (96 well plate) respectively. 24 hrs later, cells were transfected with 100 nmol/L of siRNA (or 50 nmol/L for their combinations) using Lipofectamine 2000 (*Invitrogen*) according to the manufacturer's instructions. Transfected cells were cultured at 37°C for the indicated time periods and collected and lysed for immunoblot analysis with the indicated antibodies or analyzed for cell growth by MTS analysis (*Promega*).

ShRNA constructs were based on the pKAR1/Pur plasmid (Addgene plasmid # 23105); a gift from Randy Poon [2]. Specific shRNA constructs were created by cloning the following pairs of oligonucleotides into the *BbsI* and *XbaI* sites of pKAR1/Pur: EGFR: 5′-GAGGAAAUAUGUACUACGA-3′, HER2:5′-GGACGAAUUCUGCACAAUG-3′, HER3:5′-GCAGUGGAUUCGAGAAGUG-3′ and Luciferase: 5′GCCATTCTATCCTCTAGAGGATG-3′. The shRNA-expressing plasmids were co-transfected into the NSCLC cells using Lipofectamine 2000 as described. After 48 hrs, medium was replaced with fresh medium containing 0.5 µg/ml puromycin to enrich the transfected cells. Puromycin containing medium was removed after 36 hrs and cells were grown in standard growth medium for 3 days. Cell lysates were collected and analyzed for RAS-GTP pull down and immunoblot analysis using the indicated antibodies.

Hoechst 3342/propidium iodide staining

The effects of wild-type or mutant (G12C) KRAS siRNA treatment on apoptosis and nuclear morphology in H292 cells was assessed by Hoechst 33342 and propidium iodide (PI) double fluorescent chromatin staining as described [3].

TUNEL assay

Paraffin-embedded xenografted tumor tissue (21 days post dose) was analyzed for nucleosomal DNA fragmentation using an *In Situ* Cell Death Detection Kit (*Roche*), according to the manufacturer's instructions with minor modifications.

Statistical analysis

Results are representative of three independent experiments unless stated otherwise. Values are presented as the mean ± standard error of mean (SEM). The unpaired two tailed *t*-test was utilized to compare the means of two groups. Statistical significance is reported as follows: $*P < 0.05$, $**P < 0.01$, $***P < 0.001$ and $****P < 0.0001$.

REFERENCES

1. Fleming JB, et al. Molecular consequences of silencing mutant K-ras in pancreatic cancer cells: justification for K-ras-directed therapy. Mol Cancer Res. 2005; 3:413–23.
2. Ma HT, et al. An inducible system for expression and validation of the specificity of short hairpin RNA in mammalian cells. Nucleic Acids Res. 2007; 35:e22.
3. Chen G, et al. Targeting the epidermal growth factor receptor in non-small cell lung cancer cells: the effect of combining RNA interference with tyrosine kinase inhibitors or cetuximab. BMC Med. 2012; 10:28.

Supplementary Table S1. Related to Figure 4: Summary table denoting effects of erlotinib (100 nM), pertuzumab (25 µg/ml) or combination treatment on EGFR/HER signalling in KRAS mutant NSCLC cells.

	EGFR/HER-dependent		EGFR/HER-independent	
**Erlotinib/ Pertuzumab/ Combination**	**H358**	**H23**	**A427**	**A549**
pEGFR	– / 0 / –	0 / 0 / 0	+/ +++ / ++	++ / +/ +
pHER2	0 / – / –	0 / 0 / 0	+/ +++/ ++	0 / – / –
pHER3	0 / – /–	0 / 0 / 0	+ / ++ +/ ++	+ / 0 / –
pSTAT3	0 / – /–	0 / 0 / 0	0 / – / –	0 / 0 / –
pAKT	– / –– /–––	0 / 0 / 0	0 / 0 / 0	0 / 0 / –
pERK1/2	– / –– /–––	0 / 0 / 0	0 / 0 / 0	0 / 0 / 0

Symbols (relative to vehicle conditions) are defined as follows: + : enhanced effect ; 0 : no effect ; – : decreased effect.

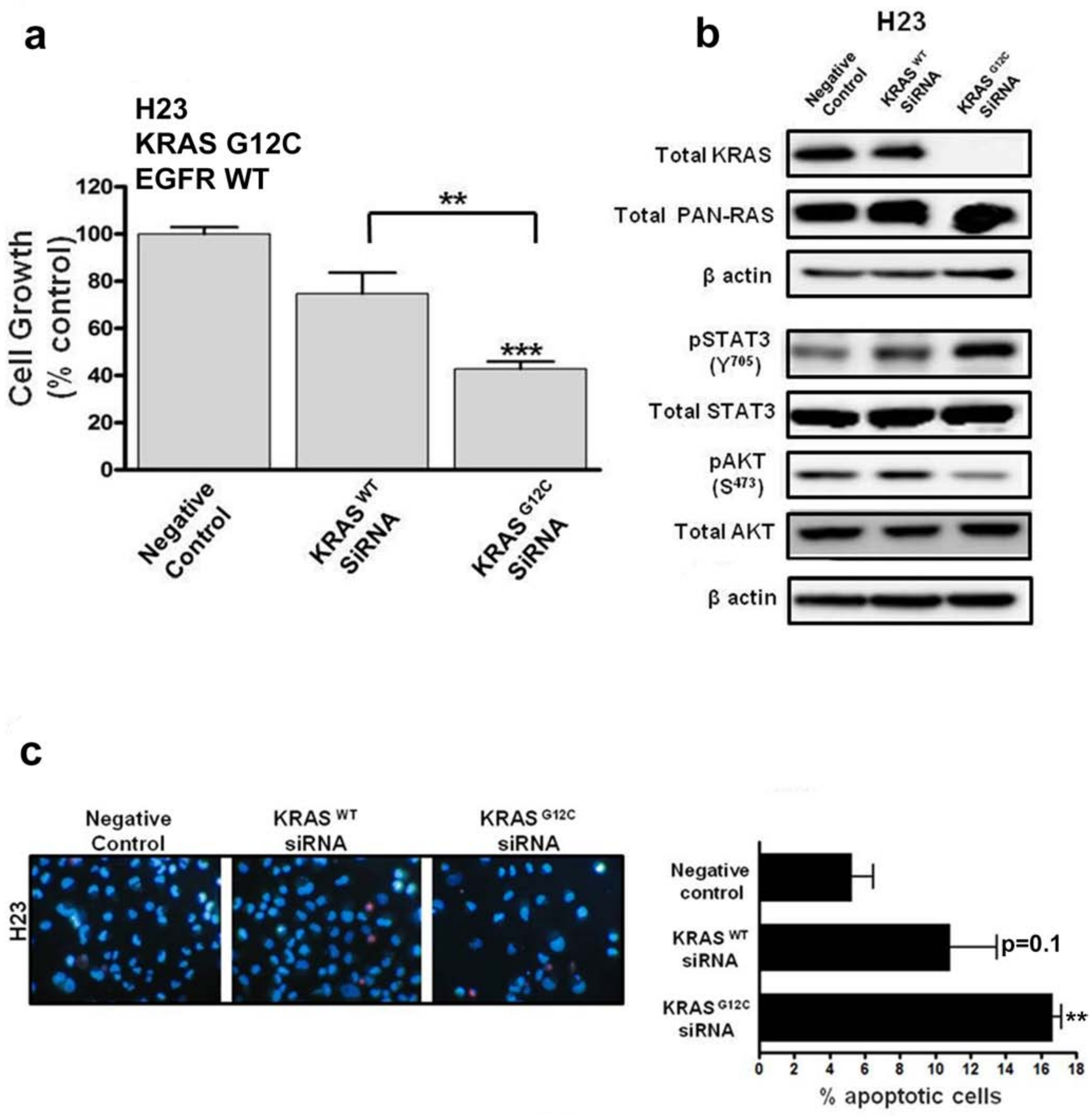

Supplementary Figure S1, related to Figure 1: Silencing oncogenic KRAS in KRAS-addicted NSCLC cells. H23 cells transiently transfected with wild-type KRAS or mutant KRAS (G12C) siRNA for 72 hrs were assessed for **a.** cell growth by MTS analysis (values are representative of mean ± SEM) and **b.** immunoblot analysis with the indicated antibodies. **c.** Cellular apoptosis was quantified by hoechst 33342 (blue) and propidium iodide (red) double fluorescent chromatin staining on cell cultures 72 hrs post siRNA transfection. Representative images of two independent experiments from 3 to 5 randomly selected microscopic fields are shown (40× magnification).

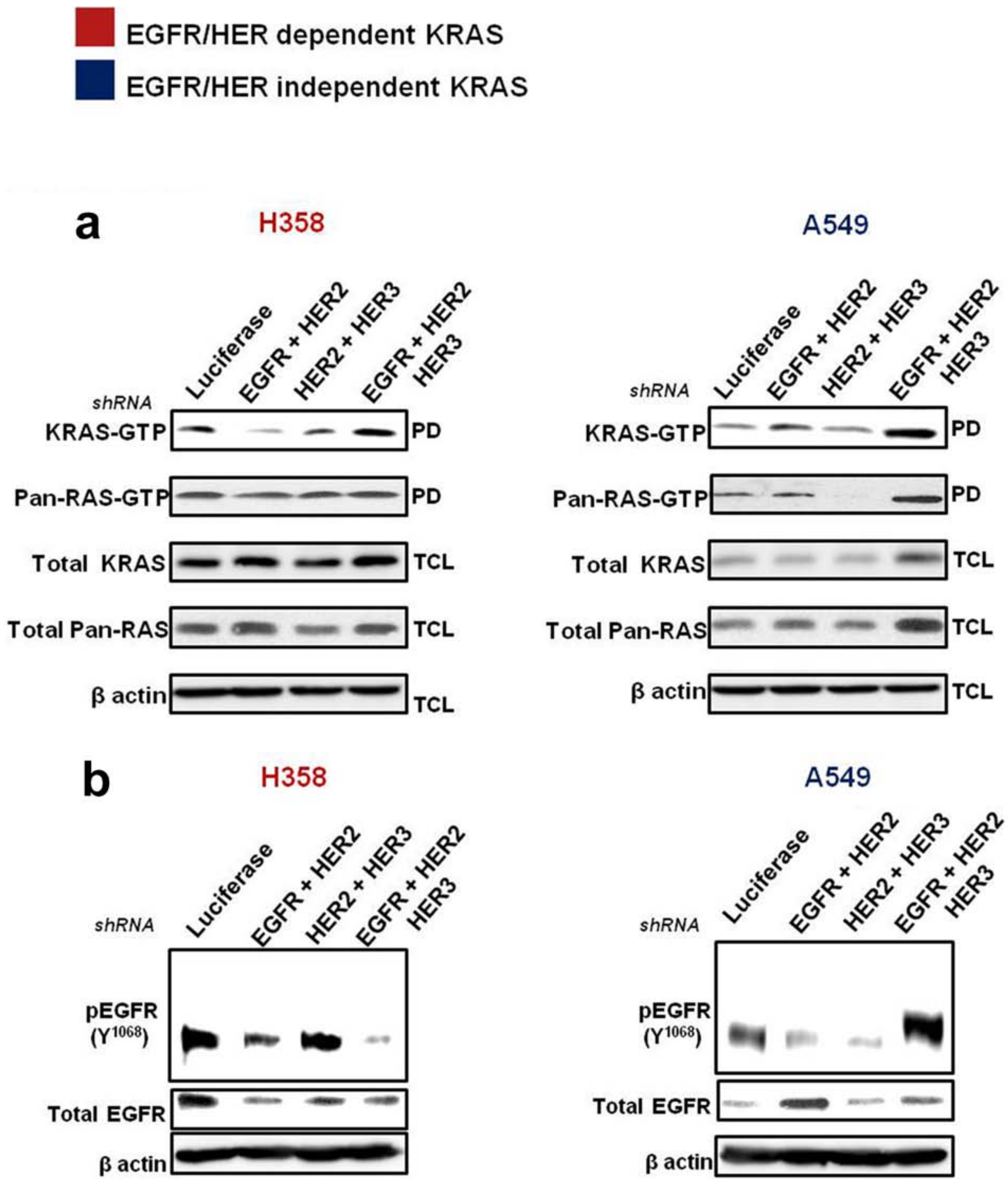

Supplementary Figure S2, related to Figure 3: Silencing EGFR/HER in mutant KRAS NSCLC cells. Cells were co-transfected with shRNA targeting Luciferase, EGFR, HER2 and HER3 and enriched with puromycin (see Materials and Methods). Ras-GTP levels were measured with a pull down (PD) assay and total cell lysates (TCL) were subjected to immunoblot analysis with the indicated antibodies **a, b**.

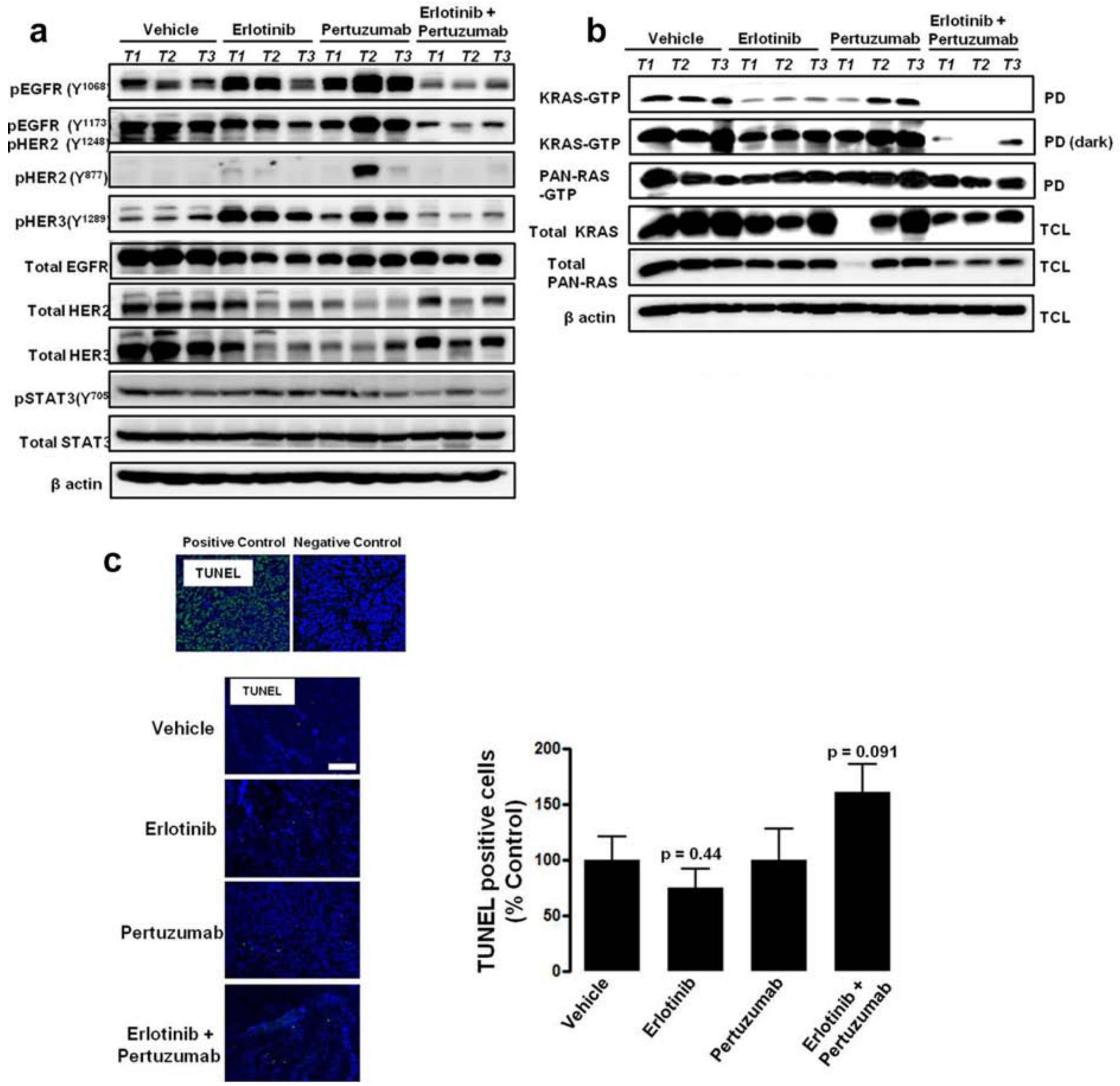

Supplementary Figure S3, related to Figure 6: Anti-tumor efficacy of sequential erlotinib/pertuzumab treatment in KRAS mutant xenografts. Mutant KRAS H358 cells were implanted subcutaneously into nude mice until tumors reached a volume of approximately 100mm^3. Xenografts (n = 8) were randomized and received vehicle,60 mg/kg erlotinib, 7 mg/kg pertuzumab or sequential combination treatment three days a week for 17 days. **a.** Total cell lysates from crude tumor tissue (T) were subjected to immunoblot analysis using the indicated antibodies. **b.** Ras-GTP levels from total cell lystates extracted from crude tumor tissue were measured with a pull-down assay (PD) by using the RAS-binding domain (RBD) of Raf-1. GTP-bound Ras, isolated from the PD and total cell lysate (TCL) subjected to immunoblot analysis are shown. Data shown are representative of three randomly selected tumorsamples from respective treatment groups. **c.** Representative TUNEL stained (DAPI counterstained) mouse tumor sections from respective treatment groups and quantification of ratio of apoptotic cells relative to vehicle control. Positive control: DNAse I treament; Negative control: label solution. Scale bar, 50 µm.
